# Knowledge and Practice of Eyelid Hygiene Among the General Population in Al-Qunfudah Governorate, Saudi Arabia: A Cross-Sectional Study

**DOI:** 10.7759/cureus.84914

**Published:** 2025-05-27

**Authors:** Safa H Alkalash, Hassan A Alzubaidi, Nawaf M Alsuhaymi, Abdulwahab A Alzubaidi, Ali N Alfaqih, Mohammed H Alothiqi, Khaled A Alfaqih

**Affiliations:** 1 Department of Community Medicine and Health Care, Umm Al-Qura University, Al-Qunfudah, SAU; 2 Department of Family Medicine, Menoufia University, Shebin Elkom, EGY; 3 Department of Family and Community Medicine, King Abdulaziz Medical City, National Guard Health Affairs, Jeddah, SAU; 4 Department of Family Medicine, Southern Ardiyah Primary Health Care Center, Makkah Health Cluster, Ministry of Health, Al-Qunfudah, SAU; 5 Emergency Department, Al-Wajh General Hospital, Tabuk Health Cluster, Ministry of Health, Tabuk, SAU; 6 Department of Family Medicine, Jeddah First Cluster Training and Academic Affairs, Ministry of Health, Jeddah, SAU; 7 Emergency Department, South Al Qunfudah General Hospital, Makkah Health Cluster, Ministry of Health, Al-Qunfudah, SAU; 8 Intensive Care Unit (ICU) Department, Al Qunfudah General Hospital, Makkah Health Cluster, Ministry of Health, Al-Qunfudah, SAU

**Keywords:** dry eyes, eyelid hygiene, knowledge, make-up use, ocular diseases, practice

## Abstract

Introduction: It is believed that ocular surface diseases (OSDs) represent a public health issue. Common disorders include blepharitis, dry eyes, and meibomian gland dysfunction (MGD). Eyelid cleanliness remains essential for preserving eyelid health and relieving ocular symptoms.

Objectives: This study aims to assess the knowledge and practice of eyelid hygiene and its associated factors among the general population in Al-Qunfudah governorate, Saudi Arabia.

Methods: A cross-sectional study was conducted on a sample of 273 adults who are above 18 years of age and live in Al-Qunfudah. The data collection tool was a validated self-administered online questionnaire, which was preceded by consent and insurance to maintain the participant's confidentiality.

Results: A total of 273 eligible participants completed the study questionnaire with a mean age of 26.9 ± 9.2 years. Exactly 196 (71.8%) were females. About 253 (92.7%) wash their face after daily activities, 208 (76.2%) wash their eyelashes, 102 (37.4%) use a cleaning solution for eyelids, and 161 (59.0%) cleaned their eyelids during the past three days. A total of 199 (72.9%) had heard about dry eye disease. Only 51 (18.7%) heard about meibomian gland dysfunction. A total of 176 (98.8%) female participants reported using eye cosmetics; 69 (39.2%) used one type. The most reported cosmetic use-associated complications included eye redness 130 (47.7%), itching 118 (43.2%), dryness 62 (22.7%), and sand sensation 42 (15.3%), while 76 (27.8%) had no effect on the eye due to the cosmetic use. Retired, those who had high incomes, and those who did not use a cleaning solution for eyelids were more aware of dry eye disease than other participants, with p-values of 0.045, 0.001, and 0.001, respectively. Those who used to wash their eyelashes knew more about meibomian gland dysfunction than those who did not (p=0.001).

Conclusion: The general population of Al-Qunfudah was well-informed about dry eye disease, the usage of eye cosmetics, and their effects on the eyes, and they had good habits of washing their faces and eyelids. On the other hand, one detrimental behavior associated with a lack of understanding of meibomian gland dysfunction is sharing cosmetic tools with other people. Lastly, patients should learn about MGD, dry eyes, and basic eyelid hygiene from ophthalmologists and primary care doctors, with a focus on low-income people. Regarding the everyday eyelid hygiene procedures that the general public is advised to follow, more research is necessary.

## Introduction

A thin transparent membrane known as the conjunctiva covers the surface of the eye as well as the inner surface of the eyelid [1]. The meibomian gland is a sebaceous gland located on the superior and inferior tarsal plates. It secretes meibum, which stabilizes the tear film and protects against microbiological agents [2,3]. The epithelial layer covering the cornea and conjunctiva is compromised by ocular surface diseases such as keratitis, conjunctivitis, and dry eye syndrome, which may negatively affect the patient's visual acuity, leading to poor quality of life [4-6].

In Saudi Arabia, the prevalence of dry eye syndrome in adults is 38.4% [7]. In the United States, it has been diagnosed in 8.1% of adult Americans, with a higher incidence in women and older individuals, while meibomian gland dysfunction prevalence is 21.2% [8]. Dry eye syndrome is primarily a chronic condition caused by insufficient and low-quality tears, as well as a loss of eye lubrication and inflammatory agents [9]. In addition to redness, fluctuating vision, contact lens sensitivity, and eye fatigue, the most typical symptoms of dry eye syndrome include a dry, gritty, sandy feeling in the eyes [10].

Women of all ages use eye cosmetics frequently; nevertheless, despite intensive testing to ensure their safety, they still have negative effects on the eye surface, such as exacerbating dry eye symptoms and causing tear film instability [11,12]. Poor eyelid hygiene can also lead to ocular surface disorders (OSDs), which can cause irreversible injury to the cornea and conjunctiva [13]. Proper management and hygiene when using eye cosmetics are crucial preventative measures to support eye health and prevent pollution, debris, and application of eye cosmetics from irritating the eyes and the occurrence of infection [12]. Traditional methods of eyelid hygiene, such as warming, massaging, and washing with face cloths and baby shampoo, have been the standard [14]. However, new eyelid-warming devices provide constant and controlled moist heat therapy, while tear supplementation through artificial tears can make eye hygiene care easier and more comfortable and encourage compliance [15]. Due to the scarcity of studies in Al-Qunfudah governorate covering ocular surface diseases and cosmetic use, the current study aimed to assess the knowledge and practice of eyelid hygiene and their associated factors among the general population in Al-Qunfudah governorate, Saudi Arabia.

## Materials and methods

Study design and setting

A descriptive cross-sectional study was conducted among adults in Al-Qunfudah, Saudi Arabia, in 2024. The study targeted all adults in Al-Qunfudah aged 18 years or more, while respondents who refused to participate or had incomplete survey answers and those who were suffering from any eye disease were excluded.

Sample size

The online sample size calculator program EPI-Info™ (CDC, Atlanta, GA) was used to estimate the sample size, with a 95% confidence level and a 5% acceptable margin of error. The general population of the Al-Qunfudah governorate consists of 300,516 individuals, and according to a prior Saudi survey, 18.4% of them had adequate knowledge about eyelid hygiene [16]. The minimum sample size was 231, and it was increased to cover the non-response rate, making the sample size 273.

Study tool

The study questionnaire was initiated by the study researchers after an intensive literature review and field expert consultation. The questionnaire was composed of 37 questions and divided into five sections (Appendix A). The first section included the socio-demographic profile, while the second section included questions about eyelid hygienic habits, the third part covered knowledge of ocular surface disease, and the fourth section was about recognition of ocular symptoms. Questions concerning the ocular cosmetics used by women were included in the fifth section, which was only meant for women to complete. At the end of the questionnaire, the participants were asked what daily eyelid care practices they employ when the symptoms arise. A group of professionals examined the study questionnaire to ensure that it was clear and had valid information. The tool's validity and reliability were evaluated in a pilot study including 15 participants; Cronbach's alpha was 0.69. The final study sample did not include the pilot sample. The study researchers posted the completed questionnaire online via social media channels.

Ethical considerations

Prior to participating in the questionnaire, each participant was given the choice to participate, and their informed consent was taken, and confidentiality of their responses was ensured, as it was only utilized for research purposes. Ethical approval was obtained from the Umm Al-Qura University Research Ethics Committee, Makkah, Saudi Arabia, with approval number (HAPO-02-K-012-2023-04-1565).

Data analysis

After being gathered and examined, the data was entered into IBM Inc.'s Statistical Package for Social Sciences version 26 (Armonk, NY, USA). All statistical methods used were two-tailed with an alpha level of 0.05, considering significance if p-values were less than or equal to 0.05. Descriptive analysis was done by prescribing frequency distribution and percentage for study categorical variables, while quantitative variables were presented as mean with standard deviation. All study sections, including demographics, eye disease knowledge, eye symptoms, and cosmetic use, were tabulated or graphed. Cross-tabulation was used to show factors associated with participants' knowledge about meibomian gland dysfunction and dry eye disease using the Pearson chi-squared test and exact probability test for small frequency distributions.

## Results

A total of 273 eligible participants completed the study questionnaire. Participants' ages ranged from 18 to 66 years, with a mean age of 26.9 ± 9.2 years. Exactly 196 (71.8%) were females, and nearly all (99.6%) were Saudi, except for one (0.4%) respondent who was non-Saudi. A total of 182 (66.7%) were single, and 91 (33.3%) were married. As for educational level, 197 (72.2%) had a university education. Exactly 164 (60.1%) were students. A total of 101 (37% of respondents) reported a monthly income of less than 5,000 SR, while 88 (32.2%) reported more than 10,000 SR (Table 1).

**Table 1:** Socio-demographic characteristics of study participants, Al-Qunfudah, Saudi Arabia (n=273) The data has been represented as N, % and Mean ± SD. SR: Riyal Saudi.

Variables	N	%
Age in years		
18-20	67	24.5
21-25	84	30.8
26-30	51	18.7
31-40	44	16.1
>40	27	9.9
Mean ± SD	26.9 ± 9.2	
Gender		
Male	77	28.2
Female	196	71.8
Nationality		
Saudi	272	99.6
Non-Saudi	1	0.4
Marital status		
Single	182	66.7
Married	91	33.3
Educational level		
Below secondary	18	6.6
Secondary	58	21.2
University/above	197	72.2
Employment		
Unemployed	50	18.3
Student	164	60.1
Employed	52	19.0
Retired	7	2.6
Monthly income		
<5,000 SR	101	37.0
5,000-10,000 SR	84	30.8
>10,000 SR	88	32.2

**Figure 1:**
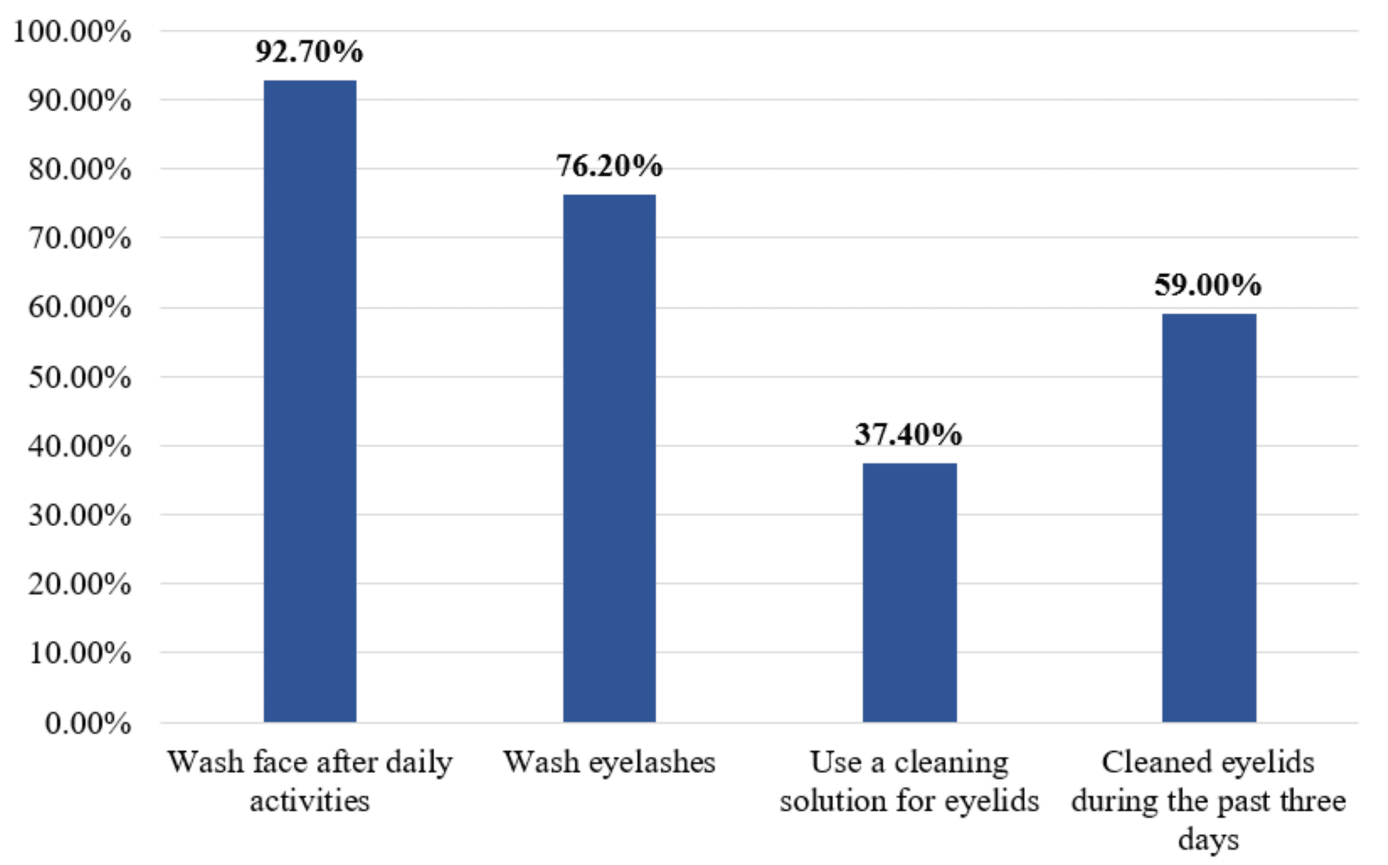
Eyelid hygienic habits among study participants, Al-Qunfudah, Saudi Arabia (n=273)

Regarding eyelid hygienic habits among study participants, 253 (92.7%) wash their face after daily activities, 208 (76.2%) wash their eyelashes, 102 (37.4%) use a cleaning solution for eyelids, and 161 (59.0%) cleaned their eyelids during the past three days (Figure 1).

A total of 199 (72.9%) had heard about dry eye disease; 86 (31.5%) said dry eye is a multifactorial disease that affects the tears and the surface of the eye, while 31 (11.4%) said it is a functional abnormality of the meibomian glands (eye gland), but 127 (46.5%) did not know about it. Only 51 (18.7%) heard about meibomian gland dysfunction, whereas 87 (31.9%) defined it as a functional abnormality of the meibomian glands, but 170 (62.3%) did not know about the disease (Table 2).

**Table 2:** Participants' knowledge about ocular surface disease and its reported eye symptoms frequency and management, Al-Qunfudah, Saudi Arabia (n=273) The data has been represented as N and %.

Variables	N	%
Background regarding dry eye disease		
Yes	199	72.9
No	74	27.1
Definition of dry eye disease		
A multifactorial disease that affects tears and the surface of the eye	86	31.5
Functional abnormalities of the meibomian glands (eye gland)	31	11.4
Opacity of the eye lens	9	3.3
Group of diseases associated with optic nerve damage	20	7.3
Don’t know	127	46.5
Background regarding meibomian gland dysfunction		
Yes	51	18.7
No	222	81.3
Definition of meibomian gland dysfunction		
Functional abnormalities of the meibomian glands (eye gland)	87	31.9
It is a multifactorial disease that affects the tears and the surface of the eye	11	4.0
Group of diseases associated with optic nerve damage	5	1.8
Don't know	170	62.3
Having a history of dry eye symptoms		
Never	50	18.3
Sometimes	172	63.0
Mostly	51	18.7
Having a history of a foreign body sensation inside eyes		
Never	84	30.8
Sometimes	157	57.5
Mostly	32	11.7
Having a history of eye fatigue		
Never	56	20.5
Sometimes	170	62.3
Mostly	47	17.2
Suffered from any uncomfortable feeling in the eye		
Never	49	17.9
Sometimes	183	67.0
Mostly	41	15.0
Eye care products used when symptoms appear		
Eye drops without a prescription	66	24.2
Prescribed eye drops	52	19.0
Cleaning the eyelid	19	7.0
Protective glasses	19	7.0
Eye warming	21	7.7
Nothing	96	35.2

Considering reported eye symptom frequency and management, 223 (81.7%) reported complaints of dry eye, 189 (69.2%) had foreign body sensation, 217 (79.5%) had eye fatigue, and 224 (82.1%) suffered from any uncomfortable feeling in the eye. As for eye care products used when symptoms appear, 66 (24.2%) use eye drops without a prescription, 21 (7.7%) use eye warming, 19 (7%) clean their eyelids, and 19 (7.0%) use protective glasses. A total of 96 (35.2%) do nothing. A total of 176 (98.8%) female participants reported using eye cosmetics; 69 (39.2%) use one type. As for used types, the most reported were mascara 154 (88%), eyeshadow 136 (77.7%), kohl 114 (65.1%), contact lenses 64 (36.6%), eyeliner 64 (36.6%), and artificial eyelashes 19 (10.9%). Mascara use was three to four times a week among 68 (43.9%), contact lenses once a month among 22 (33.3%), and artificial eyelashes less than once a month among nine (69.2%). A total of 149 (84.7%) wash their face and eyes after applying makeup, 145 (82.4%) make sure the tools are clean before using them on the eye area, and 149 (84.7%) use a cleanser to remove cosmetics. A total of 86 (48.9%) share cosmetics and tools used to apply them to the eyes with another person. As for contact lens use, 62 (93.9%) use sanitizer or wash their hands before putting on and removing contact lenses, and 57 (86.4%) keep them in lens solution (Table 3).

**Table 3:** Ocular cosmetic used among female participants, Al-Qunfudah, Saudi Arabia (n=196) The data has been represented as N and %.

Variables		N	%
Use eye cosmetics	Yes	176	89.8
	No	20	10.2
If yes, how many? (n=176)	1	69	39.2
	2	35	19.9
	3	41	23.3
	4+	31	17.6
Type of used eye cosmetics?	Mascara	154	88.0
	Eye shadow	136	77.7
	Kohl	114	65.1
	Contact lenses	64	36.6
	Eyeliner	64	36.6
	Artificial eyelashes	19	10.9
Frequency of using mascara	3 to 4 times a week	68	43.9
	1-2 times a week	38	24.5
	Once a month	29	18.7
	Less than once a month	20	12.9
Frequency of using eye shadow	3 to 4 times a week	19	14.0
	1-2 times a week	32	23.5
	Once a month	45	33.1
	Less than once a month	40	29.4
Frequency of using eyeliner	1-2 times a week	9	14.1
	Once a month	28	43.8
	Less than once a month	27	42.2
Frequency of using artificial eyelashes	1-2 times a week	3	23.1
	Once a month	1	7.7
	Less than once a month	9	69.2
Frequency of using contact lenses	3 to 4 times a week	13	19.7
	1-2 times a week	14	21.2
	Once a month	22	33.3
	Less than once a month	17	25.8
Wash face and eyes before applying makeup	Yes	149	84.7
	No	27	15.3
Make sure the tools are clean before using them on the eye area	Yes	145	82.4
	No	31	17.6
Share cosmetics and tools used to apply them to the eyes with another person	Yes	86	48.9
	No	90	51.1
Use a cleanser to remove cosmetics	Yes	149	84.7
	No	27	15.3
Use sanitizer or wash hands before putting on and removing contact lenses	Yes	62	93.9
	No	4	6.1
Solution used for contact lens	Lens solution	57	86.4
	Sterile materials	5	7.6
	Tap water	4	6.1

**Figure 2:**
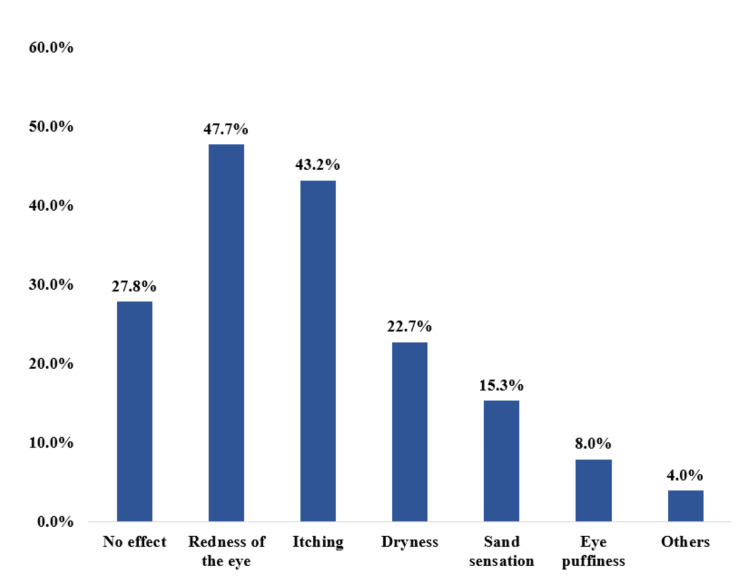
Eye effect of cosmetic use among female participants who used cosmetics, Al-Qunfudah, Saudi Arabia (n=176)

The most often reported ocular effects of cosmetic use among female participants were sand sensation 42 (15.3%), dryness 62 (22.7%), itching 118 (43.2%), and eye redness 130 (47.7%), while 76 (27.8%) had no effect on the eye due to the cosmetic use (Figure 2).

The majority of the retired individuals, six (85.7%), and those with high salaries, 73 (83.0%), had heard about dry eye syndrome, with p-values of 0.045 and 0.001, respectively. A total of 137 (80.1%) individuals who did not use an eyelid cleaning solution recalled hearing about dry eye disease, while 62 (60.8%) of those who did use it had heard of the disease (p=0.001; Table 4).

**Table 4:** Factors associated with participants knowledge about dry eye disease The data has been represented as N and %. SR: Riyal Saudi, χ^2^: chi-squared test. ^: Exact probability test; * p < 0.05 is considered statistically significant.

Variables	Knew about dry eye disease				χ^2^ test	p-value
	Yes		No			
	N	%	N	%		
Age in years					3.502	0.478
18-20	45	67.2	22	32.8		
21-25	63	75.0	21	25.0		
26-30	37	72.5	14	27.5		
31-40	31	70.5	13	29.5		
>40	23	85.2	4	14.8		
Gender					0.415	0.520
Male	54	70.1	23	29.9		
Female	145	74.0	51	26.0		
Marital status					0.454	0.500
Single	135	74.2	47	25.8		
Married	64	70.3	27	29.7		
Educational level					1.704	0.427
Below secondary	11	61.1	7	38.9		
Secondary	41	70.7	17	29.3		
University/above	147	74.6	50	25.4		
Employment					8.034^	0.045*^
Unemployed	34	68.0	16	32.0		
Student	128	78.0	36	22.0		
Employed	31	59.6	21	40.4		
Retired	6	85.7	1	14.3		
Monthly income					7.511	0.023*
<5,000 SR	66	65.3	35	34.7		
5,000-10,000 SR	60	71.4	24	28.6		
>10,000 SR	73	83.0	15	17.0		
Washing face after daily activities					11.818^	0.001*^
Yes	191	75.5	62	24.5		
No	8	40.0	12	60.0		
Washing eyelashes					2.959	0.085
Yes	157	75.5	51	24.5		
No	42	64.6	23	35.4		
Usage of a cleaning solution for eyelids					12.085	0.001*
Yes	62	60.8	40	39.2		
No	137	80.1	34	19.9		
Cleaned eyelids during the past three days					3.098	0.078
Yes	111	68.9	50	31.1		
No	88	78.6	24	21.4		

Forty-nine participants (23.6%) who wash their eyelashes learned about meibomian gland malfunction, compared to two (3.1%) who did not (p=0.001). In addition, 39 (24.2%) of those who cleaned their eyelids in the last three days heard about it, compared to 12 (10.7%) of those who did not (p=0.005; Table 5).

**Table 5:** Factors associated with participants' knowledge about meibomian gland dysfunction The data has been represented as N and %. SR: Riyal Saudi, χ^2^: chi-squared test. ^: Exact probability test, * p < 0.05 is considered statistically significant.

Variables	Knew about meibomian gland dysfunction				χ^2^ test	p-value
	Yes		No			
	N	%	N	%		
Age in years					6.655	0.155
18-20	12	17.9	55	82.1		
21-25	21	25.0	63	75.0		
26-30	7	13.7	44	86.3		
31-40	4	9.1	40	90.9		
>40	7	25.9	20	74.1		
Gender					0.815	0.367
Male	17	22.1	60	77.9		
Female	34	17.3	162	82.7		
Marital status					2.713	0.100
Single	39	21.4	143	78.6		
Married	12	13.2	79	86.8		
Educational level					4.261	0.119
Below secondary	6	33.3	12	66.7		
Secondary	7	12.1	51	87.9		
University/above	38	19.3	159	80.7		
Employment					6.132^	0.093^
Unemployed	13	26.0	37	74.0		
Student	33	20.1	131	79.9		
Employed	5	9.6%	47	90.4		
Retired	0	0.0	7	100.0		
Monthly income					1.522	0.467
<5,000 SR	16	15.8	85	84.2		
5,000-10,000 SR	15	17.9	69	82.1		
>10,000 SR	20	22.7	68	77.3		
Washing face after daily activities					1.071^	0.301^
Yes	49	19.4	204	80.6		
No	2	10.0	18	90.0		
Washing eyelashes					13.674	0.001*
Yes	49	23.6	159	76.4		
No	2	3.1	63	96.9		
Usage of a cleaning solution for eyelids					0.390	0.532
Yes	21	20.6	81	79.4		
No	30	17.5	141	82.5		
Cleaned eyelids during the past three days					7.935	0.005*
Yes	39	24.2	122	75.8		
No	12	10.7	100	89.3		

## Discussion

The current study aimed to assess the knowledge and practice of eyelid hygiene among the general population of Al-Qunfudah governorate, Saudi Arabia. This study reflected that eyelid and face hygiene was good, as most participants washed their faces and eyelashes after daily activities. Cleaning eyelids was reported among more than half of the participants, 161 (59.0%); however, about 102 (37.4%) use cleaning solution for eyelids. A previous study in Saudi Arabia revealed that 600 (54.4%) of the participants washed their eyelids regularly, and only 276 (25%) of them did not wash their face daily, which is similar to the current study findings [16]. Motoko in Japan reported poor awareness about eyelid hygiene, as only 243 (23.0%) participants reported consciously cleaning their eyelids daily [17]. Maintaining the margins of the eyelids is crucial for treating meibomian gland dysfunction (MGD), as it reduces eye discomfort and enhances the stability of the tear film [18]. The meibomian orifice, which is connected to MGD and dry eye, can get blocked by an unclean lid edge. Furthermore, Demodex, a common human ectoparasite associated with blepharitis, might proliferate due to unclean lash roots [19].

Even though most survey respondents had heard of dry eye disease, only around 86 (31.5%) of them could correctly describe it, according to the existing study. On the other hand, 51 (18.7%) of them heard about MGD. Similar results were reported by a few studies [16,20-22]; however, AlSomali et al. reported that 149 (28.5%) of their study respondents in the Eastern Saudi region were aware of dry eye, which is much lower than the estimated level in the current study [23]. In disagreement with this study's results, another Saudi study detected that few participants, 43 (8.7%), knew about dry eye disease [24].

According to the ongoing research, the most common eye symptoms mentioned were eye fatigue, dry eye, foreign body sensation, and irritation in the eye. As for eye care products used when symptoms appear, 66 (24.2%) study participants used eye drops without a prescription, and 52 (19.0 %) used eye drops with a prescription, but a smaller percentage used eye warming and cleaning the eyelid. About 96 (35.2%) of those with eye symptoms did nothing. Alhamazani et al. documented that 1,039 (94.3%) respondents reported irritation, which was the chief complaint in patients with dry eye [16].

Considering cosmetic use among the female participants, the current study showed that most of the female participants reported using eye cosmetics, where the most reported were mascara, eye shadow, kohl, contact lenses, and eyeliner, but with much less frequency, artificial eyelashes. While contact lenses, eyeshadow, and eyeliner were used once a month, mascara was used three to four times. According to research by Alhamazani et al., 841 (76.3%) of respondents were female, and 611 (72.7%) of them mentioned they used eye cosmetics [16]. Ng et al. found that 1,129 (83.0%) of their participants used eye makeup at least three times a week, with mascara being the most popular product, and 721 (53.0%) routinely used at least three different eye makeup items [12]. The current study detected that most women wash their face and eyes after applying makeup, make sure the tools are clean before using them on the eye area, and also use a cleanser to remove cosmetics. However, approximately 86 (48.9%) share eye makeup and the instruments needed to apply it with another individual. The majority of contact lens users wash their hands or use hand sanitizer before putting on, taking off, and storing their lenses in solution. This acceptable cosmetic practice demonstrates a high level of understanding regarding the impact of eye cosmetics on the eye and how they relate to OSDs. According to Motoko, the majority of the female participants in their study who applied eye cosmetics were not aware of the possibility of developing ocular surface conditions such as MGD and blepharitis [17]. Other studies have shown that public awareness of the effects of ocular cosmetics is generally low; the results of the Nigerian study indicate that respondents are aware of the impacts of ocular cosmetics but still do not have a thorough understanding of the latent consequences, which typically manifest later in life [25]. The percentage of people with dry eyes who used lenses every day was 48.8%, whereas the percentage of people who used them every year was 25%, according to a previous study conducted in Asir Region, Saudi Arabia [26].

Regarding the eye effect of cosmetic use among female participants, the most reported included eye redness, itching, dryness, and sand sensation, while 76 (27.8%) had no effect on the eye due to the cosmetic use. Similar findings were reported by another study [27]. On the other hand, Xie et al. found that in comparison to control patients who received only artificial tears and lid warming, patients who received lid margin cleaning with a deep cleaning device along with artificial tears and lid warming showed significantly better improvements in dry eye symptoms, according to a prospective study on patients with MGD-associated dry eye [15]. Mylla Boso et al. encouraged ocular surface practices without the need to discontinue glaucoma medication [28].

In this study, retired people, those who had high incomes, and those who did not use a cleaning solution for eyelids were significantly more aware of dry eye disease than other participants, with p-values of 0.045, 0.001, and 0.001, respectively. This finding is different from that acquired by AlSomali et al., who found that good knowledge is higher among young people and those who have work related to health care [23].

Considering the insightful information this study offered, a few restrictions have to be noted. Causal inferences are limited by the cross-sectional design, and biases may be introduced by the use of self-reported data. Future studies should include objective clinical assessments and take longitudinal techniques into account. Additionally, the study participants were selected as a convenience sample, which may result in a maldistribution of the target population. However, a straightforward probability sampling approach like simple random or systematic random sampling could be used in the future to get around this. Despite the previously mentioned limitations, this study highlighted eye hygiene, as its ignorance may lead to permanent disability and negatively affect the individual's quality of life.

## Conclusions

In conclusion, the current study showed good eyelid and face cleaning practices among the study participants with adequate awareness about dry eye disease, eye cosmetic use, and its ocular effects. On the other hand, poor awareness about meibomian gland dysfunction was reported with one unhealthy practice, which is sharing cosmetic tools with others. Most of the eye cosmetic users experienced one of the eye-associated complaints, such as eye redness and itching. It is suggested that ophthalmologists and primary care physicians raise patients' awareness about MGD, eye dryness, and proper eye cleanliness procedures. There is currently inadequate data in the literature to support the benefits of maintaining good eyelid hygiene in a healthy population free of MGD symptoms and clinical indications. Before we actively promote eyelid hygiene as a regular habit for the public, more research and data are required.

## Appendices

Appendix A

**Table 6:** Questionnaire to assess knowledge and practice of eyelid hygiene among Al-Qunfudah general population

Section 1: Demographics																
What is your age?																
What is your gender?																
Male					Female											
What is your nationality?																
Saudi					Non-Saudi											
What is your residence?																
Al-Qunfudah					Villages surrounding Al-Qunfudah						Outside Al-Qunfudah					
What is your education level?																
Below secondary					Secondary						University/above					
What is your marital status?																
Single					Married											
What is your employment?																
Employed					Unemployed				Student					Retired		
What is your monthly income in Saudi Riyal?																
<5,000																
5,000-10,000																
>10,000																
Section 2: Face and eyelid hygiene habits																
Do you wash your face after daily activities?																
Yes					No											
Do you wash your eyelashes?																
Yes					No											
Do you use a cleaning solution for your eyelids?																
Yes					No											
Have you cleaned your eyelids in the past three days?																
Yes					No											
Section 3: Awareness of dry eye disease and meibomian gland dysfunction																
Have you heard of dry eye disease?																
Yes						No										
Choose one correct statement about dry eye disease:																
A multi-factorial disease that affects the tear film and eye surface																
Caused by functional abnormalities of the meibomian glands																
A group of diseases associated with optic nerve damage																
Opacification of the lens																
Have you heard of meibomian gland dysfunction ?																
Yes							No									
Choose one correct statement about meibomian gland dysfunction :																
Functional abnormality of the meibomian glands																
A multi-factorial disease affecting tear film and ocular surface																
A group of diseases associated with optic nerve damage																
Section 4: History of eye symptoms and management																
How often do you have a dry eye symptom?																
Never				Sometimes								Mostly				
How often do you have a foreign body sensation inside eyes?																
Never				Sometimes								Mostly				
How often do you have eye fatigue?																
Never				Sometimes								Mostly				
How often do you suffer from uncomfortable feeling in the eye?																
Never				Sometimes								Mostly				
What do you do when having eye symptoms?																
Use eye drops without a prescription																
Use prescribed eye drops																
Clean the eyelid																
Wear protective eyeglasses																
Perform eye warming																
Nothing																
Section 5: Use of eye makeup (for females)																
Do you use eye makeup products?																
Yes							No									
How many eye makeup products do you use in a day?																
One	Two		Three				Four or more			None						
What type do you usually use?																
Eye shadow	Eyeliner		Mascara				Artificial eyelashes			Contact lenses			Kohl			None
How often do you use mascara?																
Never		Less than once a month		Once a month				1-2 times a week				3-4 times a week			Daily	
How often do you use eye shadow?																
Never		Less than once a month		Once a month				1-2 times a week				3-4 times a week			Daily	
How often do you use eyeliner?																
Never		Less than once a month		Once a month				1-2 times a week				3-4 times a week			Daily	
How often do you use artificial eyelashes?																
Never		Less than once a month		Once a month				1-2 times a week				3-4 times a week			Daily	
How often do you use contact lenses?																
Never		Less than once a month		Once a month				1-2 times a week				3-4 times a week			Daily	
What solution used for contact lenses?																
Lens solution																
Sterile materials																
Tap water																
Do you wash your face and eyes before applying makeup?																
Yes								No								
Do you make sure the tools used around your eyes are clean?																
Yes								No								
Do you share your personal eye makeup with others?																
Yes								No								
Do you wash your hands or use sanitizer before wearing and removing contact lenses?																
Yes								No								
Do you use a cleanser to remove cosmetics?																
Yes								No								
Have you ever experienced any eye effects due to cosmetic product use?																
Yes								No								
If yes, which effects did you experience?																
Tearing																
Itching																
Redness																
Skin rash around the eye																
Swelling of the eye																
Dry eyes																
Sandy sensation in the eye																
